# Identification of Novel Bisbenzimidazole Derivatives as Anticancer Vacuolar (H^+^)-ATPase Inhibitors †

**DOI:** 10.3390/molecules22091559

**Published:** 2017-09-16

**Authors:** Renukadevi Patil, Arpita Kulshrestha, Anjali Tikoo, Sara Fleetwood, Gajendra Katara, Bala Kolli, William Seibel, Alice Gilman-Sachs, Shivaputra A. Patil, Kenneth D. Beaman

**Affiliations:** 1 Pharmaceutical Sciences Department, College of Pharmacy, Rosalind Franklin University of Medicine and Science, North Chicago, IL 60064, USA; renukadevi.patil@rosalindfranklin.edu; 2 Department of Microbiology and Immunology, Chicago Medical School, Rosalind Franklin University of Medicine and Science, North Chicago, IL 60064, USA; arpita.kulshrestha@rosalindfranklin.edu (A.K.); anjali.tikoo@rosalindfranklin.edu (A.T.); sara.fleetwood@rosalindfranklin.edu (S.F.); gajendra.katara@rosalindfranklin.edu (G.K.); bala.kolli@rosalindfranklin.edu (B.K.); alice.gilman-sachs@rosalindfranklin.edu (A.G.-S.); 3 Division of Oncology, Cincinnati Children’s Hospital Medical Center, 3333 Burnet Avenue, Cincinnati, OH 45229, USA; william.seibel@cchmc.org

**Keywords:** vacuolar (H^+^)-ATPase (V-ATPase), inhibitor, bisbenzimidazole, breast cancer, ovarian cancer, antiproliferative activity, proton (H^+^) pump activity

## Abstract

The vacuolar (H^+^)-ATPases (V-ATPases) are a family of ATP-driven proton pumps and they have been associated with cancer invasion, metastasis, and drug resistance. Despite the clear involvement of V-ATPases in cancer, the therapeutic use of V-ATPase-targeting small molecules has not reached human clinical trials to date. Thus, V-ATPases are emerging as important targets for the identification of potential novel therapeutic agents. We identified a bisbenzimidazole derivative (**V**) as an initial hit from a similarity search using four known V-ATPase inhibitors (**I**–**IV**). Based on the initial hit (**V**), we designed and synthesized a focused set of novel bisbenzimidazole analogs (**2a**–**e**). All newly prepared compounds have been screened for selected human breast cancer (MDA-MB-468, MDA-MB-231, and MCF7) and ovarian cancer (A2780, Cis-A2780, and PA-1) cell lines, along with the normal breast epithelial cell line, MCF10A. The bisbenzimidazole derivative (**2e**) is active against all cell lines tested. Remarkably, it demonstrated high cytotoxicity against the triple-negative breast cancer (TNBC) cell line, MDA-MB-468 (IC_50_ = 0.04 ± 0.02 μM). Additionally, it has been shown to inhibit the V-ATPase pump that is mainly responsible for acidification. To the best of our knowledge the bisbenzimidazole pharmacophore has been identified as the first V-ATPase inhibitor in its class. These results strongly suggest that the compound **2e** could be further developed as a potential anticancer V-ATPase inhibitor for breast cancer treatment.

## 1. Introduction

The vacuolar (H^+^)-ATPases (V-ATPases) are a family of ATP-driven proton pumps that couple ATP hydrolysis with the translocation of protons across membranes, and they are present in both the intracellular and cell surface membranes of eukaryotes. The V-ATPase proton pump is a macromolecular complex composed of at least 14 subunits organized into two functional domains, V1 and V0. The domain V1 is responsible for ATP hydrolysis and V0 provides the transmembrane proton channel [1,2,3,4,5]. The V-ATPase plays a major role in the regulation of cellular pH conditions, and it has been associated with cancer invasion, metastasis, and drug resistance [6,7,8,9]. Despite knowing the clear involvement of V-ATPases in cancer, the therapeutic use of V-ATPase-targeting small molecules has not reached the clinic currently. Thus, V-ATPases are emerging as important targets for the identification of potential novel chemo therapeutic agents [10]. Macrolide antibiotics such as bafilomycin and concanamycin potently inhibit V-ATPases [11,12,13,14,15], but their use is complicated by adverse side effects on other targets. Few unnatural small molecule drugs have been explored as V-ATPase inhibitors, but the need for compounds that specifically target the mechanism(s) responsible for the low pH of tumors is growing. The development of useful V-ATPase inhibitors has been limited, even though huge efforts by both pharmaceutical industry and academic medicinal chemists are made, because of the complicated chemical structures of existing natural inhibitors. 

**Figure 1:**
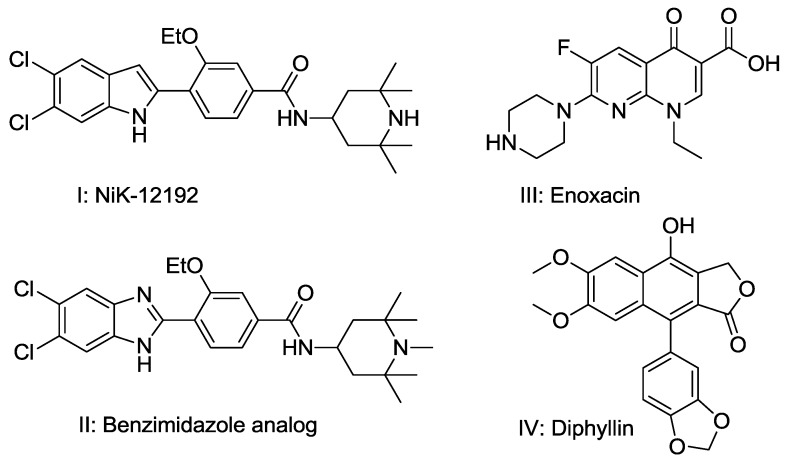
Structures of literature reported small molecule vacuolar (H^+^)-ATPase (V-ATPase) inhibitors.

Novel small molecules with defined mechanisms of inhibition against V-ATPase are needed to evaluate their therapeutic potential in cancer. Among many mechanisms that regulate the tumor microenvironment, V-ATPases are especially significant because they can be inhibited by proton pump inhibitors. The easiest method to design and develop new V-ATPase inhibitors is the screening processes. Drug screening can be performed using various in vitro assays. In vitro drug screening provides a quick, cost-effective method for the selection of potent V-ATPase inhibitors. Planning the course of effective screening mainly involves the selection of a proper set of compounds, built in-house or obtained from commercially available libraries. In an effort to identify the new small molecule V-ATPase inhibitors, we used four known small molecule V-ATPase inhibitors to perform similarity searches against the University of Cincinnati, Drug Discovery Center (UC DDC) Library, consisting of 362,000 compounds, and the National Cancer Institute (NCI) Small Molecule Repository (SMR), with 263,365 compounds. Similarity searches were performed in Accelrys’ Pipeline Pilot using 13 fingerprint types in parallel [16,17,18]. Compounds I and II were developed from the most promising member of the indole class of V-ATPase inhibitors, (2*Z*,4*E*)-5-(5,6-dichloro-2-indolyl)-2-methoxy-*N*-(1,2,2,6,6-pentamethylpiperidin-4-yl)-2,4-pentadienamide (SB 242784) [19,20]. Compound III, Enoxacin, is a fluoroquinolone antibiotic that was identified by structure-based virtual screening [21]. The natural product Diphyllin (IV) functions as a potent inhibitor of osteoclastic V-ATPase [22] (Figure 1).

## 2. Results and Discussion

### 2.1. Chemistry

#### 2.1.1. Similarity Search

**Figure 2:**
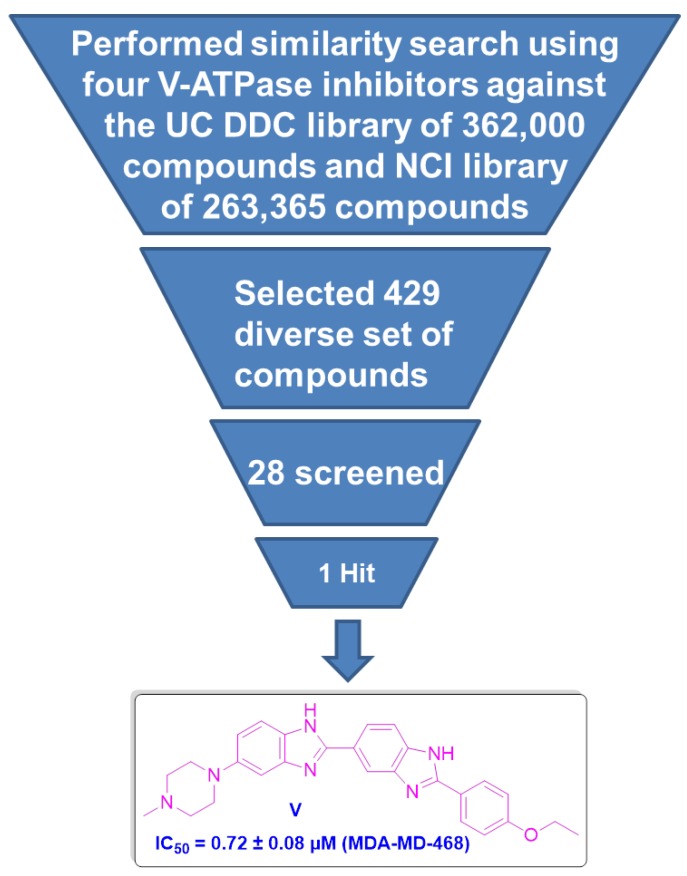
Identification of an initial hit by similarity search using known V-ATPase inhibitors.

We performed the similarity search process based on the four compounds listed above (**I**–**IV**; Figure 1). Initially, we selected approximately 429 compounds for the study. Subsets of 28 compounds were manually selected for anticancer screening using Lipinski’s rules and a visual perception of relatedness to the compounds of interest. All 28 compounds were screened for a single human breast cancer cell line, MDA-MB-231, and identified the bisbenzimidazole derivative (compound **V**) as an initial hit (Figure 2). The compound **V** served as a starting point in our program for finding potent new anticancer V-ATPase agents by designing and synthesizing a small set of bisbenzimidazole analogs. These new analogs were screened for antiproliferative activity using a panel of breast and ovarian cancer cell lines. The selected potent anticancer compounds were measured for proton pump activity. 

#### 2.1.2. Chemical Synthesis 

**Scheme 1:**
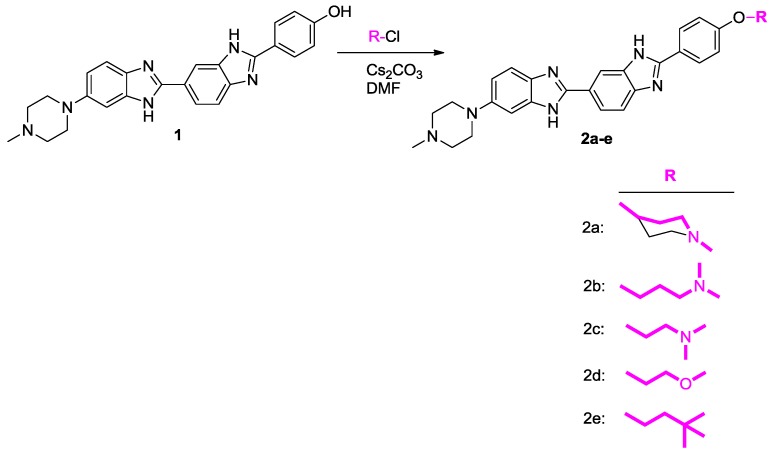
Synthesis of bisbenzimidazole analogs **2a**–**e**.

We quickly developed a fast and efficient synthetic procedure to prepare novel bisbenzimidazole analogs using the one pot procedure. A general synthesis of bisbenzimidazole derivatives (**2a**–**e**) has been achieved by the condensation of 4-(6-(4-methylpiperazin-1-yl)-1*H*,3′*H*-[2,5′-bibenzo[d]imidazol]-2′-yl)phenol (compound **1**) with appropriate substituted alkyl halides in the presence of cesium carbonate in dimethyl formamide (DMF) at reflux temperature (Scheme 1). The crude residue was subjected to flash column chromatography to obtain pure bisbenzimidazole derivatives (**2a**–**e**) using methanol and chloroform (3:7). 

### 2.2. Biology

#### 2.2.1. Antiproliferative Activity

Our research group is extensively involved in studying the role of vacuolar (H^+^)-ATPase in breast as well as ovarian cancer cell lines [23,24,25,26]. We examined the antiproliferative activity of the novel bisbenzimidazole derivatives (**2a**–**e**) in selected human breast cancer cell lines, MDA-MB-231 (ATCC, HTB-26), MDA-MB-468 (ATCC, HTB-132), MCF-7 (ATCC, HTB-22), and the normal breast epithelial cell line, MCF10A. We also used the cisplatin-sensitive A2780, PA-1 (ATCC, CRL 1572), and the cisplatin-resistant Cis-A2780 ovarian cancer cell lines. A potent V-ATPase inhibitor, Bafilomycin A1 (Baf A1), was used as the positive control [27,28]. We employed the CellTiter 96 AQueous One solution cell proliferation assay (MTS; Promega) [29,30] to measure the in vitro cell viability of the cells. 

**Figure 3:**
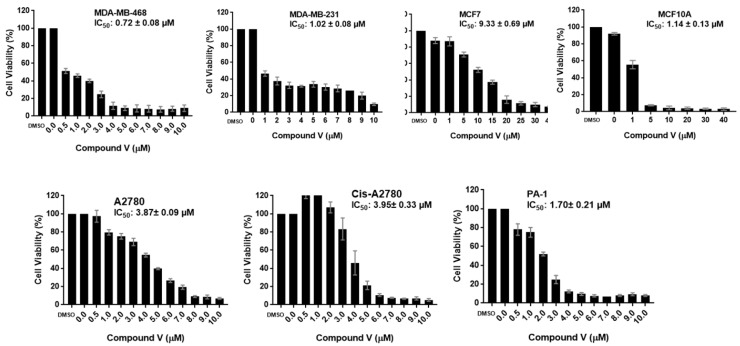
The cell viability (%) relative to untreated control cells of selected breast and ovarian cancer cell lines and breast normal (MCF10 A) cell line following the exposure of various concentrations of compound **V** for 48 h.

**Figure 4:**
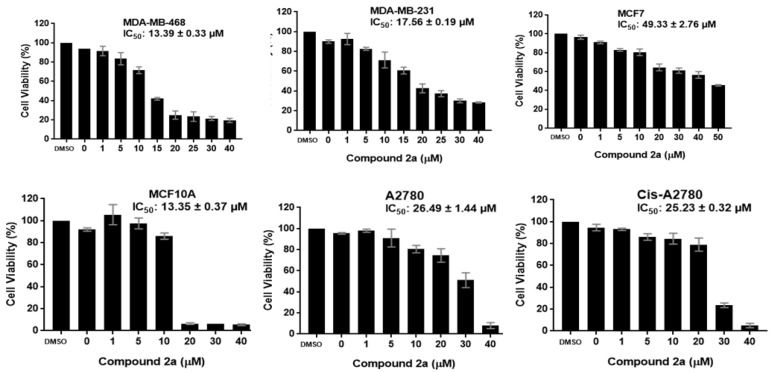
The cell viability (%) relative to untreated control cells of selected breast and ovarian cancer cell lines and breast normal (MCF10 A) cell line following the exposure of various concentrations of compound **2a** for 48 h.

**Figure 5:**
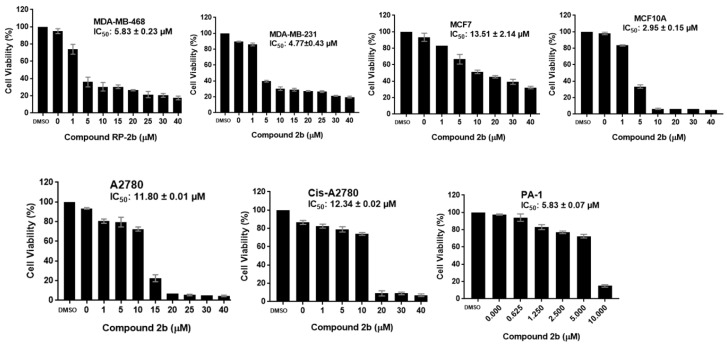
The cell viability (%) relative to untreated control cells of selected breast and ovarian cancer cell lines and breast normal (MCF10 A) cell line following the exposure of various concentrations of compound **2b** for 48 h.

**Figure 6:**
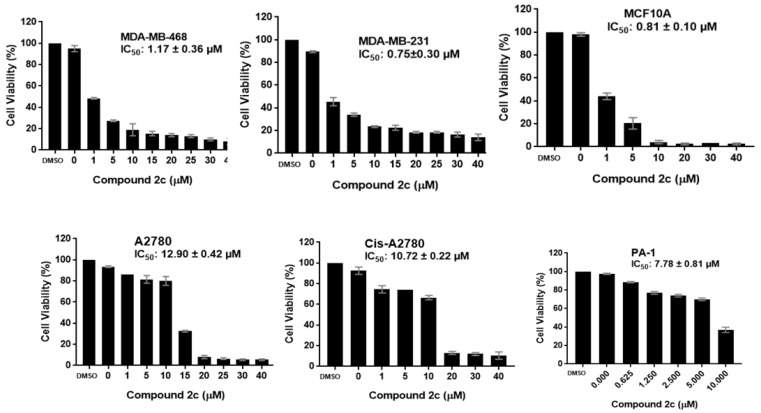
The cell viability (%) relative to untreated control cells of selected breast and ovarian cancer cell lines and breast normal (MCF10 A) cell line following the exposure of various concentrations of compound **2c** for 48 h.

**Figure 7:**
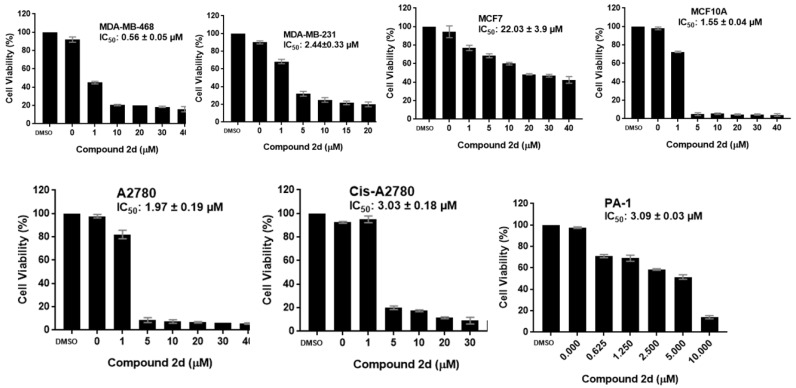
The cell viability (%) relative to untreated control cells of selected breast and ovarian cancer cell lines and breast normal (MCF10 A) cell line following the exposure of various concentrations of compound **2d** for 48 h.

**Figure 8:**
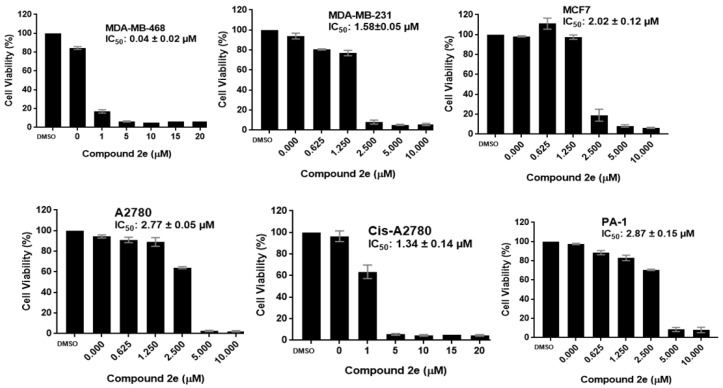
The cell viability (%) relative to untreated control cells of selected breast and ovarian cancer cell lines following the exposure of various concentrations of compound **2e** for 48 h.

**Figure 9:**
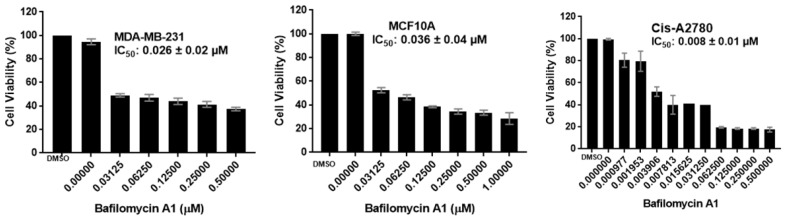
The cell viability (%) relative to untreated control cells of breast cancer (MDA-MB-231), breast normal (MCF10A) cell lines, and ovarian cancer (Cis-A2780) cell line following the exposure of various concentrations of standard Baf A1 for 48 h.

Our initial hit (compound **V**, Figure 3) demonstrated a single digit micoromolar activity (IC_50_ range 0.72 ± 0.08–9.33 ± 0.69 μM) towards all of the cell lines tested. The newly prepared compounds (**2a**–**e**) have shown encouraging antiproliferative activity (Figure 4, Figure 5, Figure 6, Figure 7 and Figure 8) in comparison with the positive control, Baf A1 (Figure 9) (Table 1).

Interestingly, our hit (compound **V)** demonstrated a high cytotoxicity towards the triple-negative breast cancer (TNBC) cell line, MDA-MB-468 (IC_50_ = 0.72 ± 0.08 μM). We used a focused set of compounds to quickly obtain the structure-activity relationship (SAR) study. Our effort to replace the ethyl group from the hit compound (**V**) with hydrophilic (**2a**–**d**) and hydrophobic (**2e**) end groups with a different carbon linker yielded the highly potent anticancer V-ATPase inhibitor. The hydrophilic end group compounds with three carbon linkers, compounds **2a** (cyclic amine) and **2b** (open chain amine), have shown modest anticancer activity. The two carbon linker hydrophilic end group compounds, **2c** and **2d,** have displayed very good cytotoxicity towards MDA-MB-231 (IC_50_ = 0.75 ± 0.30 μM)/MDA-MB-468 (IC_50_ = 1.17 ± 0.36 μM) and MDA-MB-231 (IC_50_ = 2.44 ± 0.33 μM)/MDA-MB-468 (IC_50_ = 0.56 ± 0.05 μM), respectively. Unfortunately, these compounds lack selectivity towards the normal breast epithelial cell line, MCF10A. We planned to introduce the hydrophobic end group with the two carbon linker (compound **2e**), since the two carbon linker compounds were better than the three carbon linker compounds in the case of the hydrophilic end group compounds. To our surprise, compound **2e** demonstrated a selective cytotoxicity towards only the MDA-MB-468 cell line (IC_50_ = 0.04 ± 0.02 μM), but it is not selective for other breast cancer cell lines. It is nearly 40 times less toxic than the normal breast epithelial cell line, MCF10A (IC_50_ = 1.62 ± 0.14 μM). The compound **2e** showed good antiproliferative activity towards the cisplatin-resistant ovarian cancer cell line (Cis-A2780: IC_50_ = 1.34 ± 0.14 μM), whereas it showed moderate activity against cisplatin-sensitive ovarian cancer cell lines (A2780: IC_50_ = 2.77 ± 0.05 μM; PA-1: IC_50_ = 2.87 ± 0.15 μM). This indicates that it could be further developed for resistant cell lines to overcome drug resistance. With our quick SAR studies, we identified the highly potent anticancer agent, compound **2e**.

#### 2.2.2. Proton (H^+^) Pump Activity 

**Figure 10:**
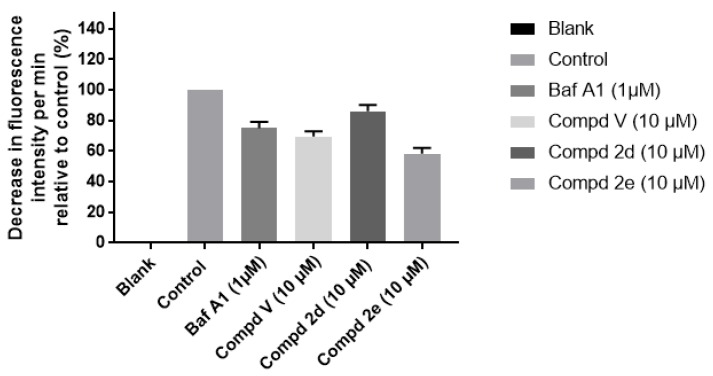
Effect of V-ATPase inhibitors on proton pump activity. Cells (MDA-MB-231) were subjected to acridine orange (AO) fluorescence quenching assay in the presence and absence of Baf A1 (1 μM) and potent compounds (**V**, **2d**, and **2e**; 10 μM**)**. Fluorescence intensity at an excitation of 495 nm and an emission of 540 nm was quantified. Decrease in fluorescence intensity per min was calculated as percentage relative to control (mean ± SD; *n* = 2).

Since our hit compound (**V**) has been identified by the literature known V-ATPases, we hypothesized that these newly synthesized bisbenzimidazole derivatives (**2a**–**e**) will work as proton pump inhibitors (PPIs). To neutralize the acidic microenvironment, PPIs have been used to target the V-ATPase pumps present on the cell membrane. PPIs have been shown to be highly effective at inhibiting V-ATPases in vitro and displayed efficacy in animal models [31,32,33,34]. Therefore, we selected efficacious anticancer agents for the proton pump activity. We performed the standard proton pump activity using the acridine orange (AO) fluorescence quenching method [35]. We selected two highly efficacious compounds (**2d** and **2e**) and our initial hit (**V**) for proton pump activity. Baf A1 is a known V-ATPase inhibitor and is used as a positive control. The proton pump activity of cells was measured as a decrease in the fluorescence intensity per min. The activity of cells in the presence of Baf A1 and the potent compounds (**V**, **2d,** and **2e)** was calculated as a percentage of the untreated control. The values represented are mean values of duplicate measurements (Figure 10). Baf A1 (1 μM) showed a 25% inhibition of the H^+^-ATPase activity, whereas our hit compound (**V**) exhibited 30% at 10 μM. Our most efficacious anticancer compounds, (**2d**) and (**2e**), have shown 14% and 42% inhibition, respectively, at 10 μM. 

## 3. Experimental Section

### 3.1. Chemical General Information

All reagents were purchased from Sigma-Aldrich Chemical Co. (St. Louis, MO, USA), and Combi-Blocks, Inc. (San Diego, CA, USA) and were used without further purification. Compound **V** was obtained from the Drug Synthesis and Chemistry Branch, Developmental Therapeutic Program, Division of Cancer Treatment and Diagnosis, National Cancer Institute (NCI, Bethesda, MD, USA). The reactions were carried out in an argon atmosphere. Routine thin-layer chromatography (TLC) was performed on aluminum-backed Uniplates (Analtech, Newark, DE, USA). Melting points were determined on a Stuart™ melting point apparatus SMP10 (Sigma-Aldrich) and are uncorrected. ^1^H and ^13^C nuclear magnetic resonance (NMR) spectra were determined in DMSO-*d*_6_/or MeOD at 400 MHz and 100 MHz, respectively, using Agilent 400 MHz Spectrometer (Agilent Technologies, Santa Clara, CA, USA). Chemical shifts are reported as shifts (δ) in parts per million (ppm) relative to TMS. Mass spectra were collected on a Shimadzu LCMS-2020 electrospray/ion trap instrument (Shimadzu Scientific Instruments, Columbia, MD, USA) in positive modes. High Resolution Mass Spectrometry (HRMS) for **2a**–**e** was carried out at the University of Illinois Mass Spectrometry Lab in the School of Chemical Sciences (Urbana, IL, USA). Yields refer to purified products.

### 3.2. Synthesis

General Procedure for the Synthesis of Bisbenzimidazole Derivatives (**2a**–2**e**):

To a suspension of mixture of 2-[2-(4-hydroxyphenyl)-6-benzimidazoyl]-6-(1-methyl-4-piperazyl)benzimidazole trihydrochloride (compound **1**; 0.375 mmol, 0.200 g) in anhydrous dimethyl formamide (10 mL) was added cesium carbonate (1.873 mmol, 0.610 g) followed by the desired substituted alkyl halides (0.562 mmol), and the reaction mixture was refluxed overnight. Next day, the mixture was cooled, extracted with ethyl acetate (100 mL), and washed with water. The ethyl acetate layer was dried over anhydrous sodium sulfate and the organic solvent was evaporated under reduced pressure. The crude residue was purified by flash chromatography to obtain the pure bisbenzimidazole derivatives (**2a**–**e**) using methanol and chloroform (3:7). 

*6-(4-Methylpiperazin-1-yl)-2'-(4-((1-methylpiperidin-4-yl)oxy)phenyl)-1H,3'H-2,5'-bibenzo[d]imidazole* (**2a**) Yield: 12%, brown solid powder. m.p.: 165–166 °C; ^1^H-NMR (400 MHz, MeOD) δ 2.06–2.18 (m, 4H, 2× CH_2_), 2.55 (s, 3H, N-CH_3_), 2.70 (s, 3H, N-CH_3_), 2.89-3.00 (m, 6H, 3× CH_2_), 3.19–3.28 (m, 6H, 3× CH_2_), 4.74 (bs, 1H, CH), 7.06-7.09 (m, 1H, Ar-H), 7.18 (d, *J* = 8.8 Hz, 3H, Ar-H), 7.53 (d, *J* = 8.8 Hz, 1H, Ar-H), 7.72 (d, *J* = 8.4 Hz, 1H, Ar-H), 7.97 (d, *J* = 8.0 Hz, 1H, Ar-H), 8.09 (d, *J* = 8.4 Hz, 2H, Ar-H), 8.29 (s, 1H, Ar-H); MS (ESI): *m*/*z* 522.6 [M + H]^+^; HRMS (ESI): *m*/*z* calcd for C_31_H_35_N_7_O [M + H]^+^ 522.2981; obsd 522.2971.

*N,N-Dimethyl-3-(4-(6-(4-methylpiperazin-1-yl)-1H,3'H-*[2,5'-bibenzo[d]*imidazol]-2'-yl)phenoxy)propan-1-amine* (**2b**). Yield: 15%, brown solid powder. m.p.: 171–172 °C; ^1^H-NMR (400 MHz, DMSO-*d_6_*) δ 1.90 (quint, *J* = 6.4, 7.2 Hz, 2H, CH_2_), 2.20 (s, 6H, N(CH_3_)_2_), 2.24 (s, 3H, N-CH_3_), 2.44 (t, *J* = 6.8 Hz, 2H, CH_2_), 3.08–3.16 (m, 4H, 2× CH_2_), 3.36–4.02 (m, 4H, 2× CH_2_), 4.09 (t, *J* = 6.8 Hz, 2H, CH_2_), 6.91–6.95 (m, 2H, Ar-H), 7.12 (d, *J =* 8.4 Hz, 2H, Ar-H), 7.36–7.49 (m, 1H, Ar-H), 7.58–7.72 (m, 1H, Ar-H), 7.95–8.03 (m, 1H, Ar-H), 8.14 (d, *J =* 8.8 Hz, 2H, Ar-H), 8.20–8.33 (m, 1H, Ar-H), 12.60 (bs, 1H, NH), 12.96 (bs, 1H, NH); MS (ESI): *m*/*z* 510.6 [M + H]^+^; HRMS (ESI): *m*/*z* calcd for C_30_H_35_N_7_O [M + H]^+^ 510.2984; obsd 510.2983.

*N,N-Dimethyl-2-(4-(6-(4-methylpiperazin-1-yl)-1H,3'H-*[2,5'-bibenzo[d]*imidazol]-2'-yl)phenoxy)ethanamine* (**2c**). Yield: 13%, yellow solid powder. m.p.: 197–198 °C; ^1^H-NMR (400 MHz, DMSO-*d*_6_) δ 2.44 (s, 9H, N(CH_3_)_3_), 2.65 (t, *J =* 5.6 Hz, 2H, CH_2_), 3.10–3.13 (m, 4H, 2× CH_2_), 3.34–3.39 (m, 4H, 2× CH_2_), 4.13 (t, *J =* 5.6 Hz, 2H, CH_2_), 6.91–6.93 (m, 2H, Ar-H), 7.12 (d, *J =* 8.8 Hz, 2H, Ar-H), 7.38–7.46 (m, 1H, Ar-H), 7.65 (d, *J =* 8.4 Hz, 1H, Ar-H), 7.98 (d, *J =* 8.4 Hz, 1H, Ar-H), 8.15 (d, *J =* 8.8 Hz, 2H, Ar-H), 8.27 (s, 1H, Ar-H), 12.60 (brs, 1H, NH), 12.96 (brs, 1H, NH); MS (ESI): *m*/*z* 496.4 [M + H]^+^; HRMS (ESI): *m*/*z* calcd for C_29_H_33_N_7_O [M + H]^+^ 496.2825; obsd 496.2820.

*2'-(4-(2-Methoxyethoxy)phenyl)-6-(4-methylpiperazin-1-yl)-1H,3'H-2,5'-bibenzo[d]imidazole* (**2d**). Yield: 22%; yellow solid powder; m.p.: 208–209 °C; ^1^H-NMR (400 MHz, MeOD) δ 2.35 (s, 3H, N-CH_3_), 2.62–2.65 (m, 4H, 2× CH_2_), 3.16–3.18 (m, 4H, 2× CH_2_), 3.39 (s, 3H, OCH_3_), 3.68–3.70 (m, 2H, CH_2_), 4.05–4.08 (m, 2H, CH_2_), 6.98–7.00 (m, 3H, Ar-H), 7.07 (d, *J =* 2.0 Hz, 1H, Ar-H), 7.46 (d, *J =* 8.8 Hz, 1H, Ar-H), 7.52 (d, *J =* 8.8 Hz, 1H, Ar-H), 7.57–7.58 (m, 1H, Ar-H), 7.95 (d, *J =* 9.2 Hz, 2H, Ar-H), 8.16 (s, 1H, Ar-H); ^13^C-NMR (100 MHz, MeOD) δ 44.59, 50.25, 54.68, 57.85, 67.05, 70.63, 100.75, 114.57, 114.90, 120.98, 121.65, 124.17, 128.10, 134.5, 138.0, 148.06, 152.24, 153.70, 160.79, 171.56; MS (ESI): *m*/*z* 483.5 [M + H]^+^; HRMS (ESI): *m*/*z* calcd for C_28_H_30_N_6_O_2_ [M + H]^+^ 483.2508; obsd 483.2498.

*2'-(4-(3,3-Dimethylbutoxy)phenyl)-6-(4-methylpiperazin-1-yl)-1H,3'H-2,5'-bibenzo[d]imidazole* (**2e**). Yield: 16%; pale yellow solid powder; m.p.: 247–248 °C; ^1^H-NMR (400 MHz, DMSO-*d*_6_) δ 0.98 (s, 9H, (CH_3_)_3_), 1.70 (t, *J =* 7.2 Hz, 2H, CH_2_), 2.24 (s, 3H, N-CH_3_), 3.12–3.30 (m, 8H, 4× CH_2_), 4.11 (t, *J =* 7.2 Hz, 2H, CH_2_), 6.91-6.96 (m, 2H, Ar-H), 7.13 (d, *J =* 7.6 Hz, 2H, Ar-H), 7.58-7.72 (m, 1H, Ar-H), 7.28–7.49 (m, 1H, Ar-H), 7.94–8.02 (m, 1H, Ar-H), 8.13 (dd, *J =* 2.8, 3.2 Hz, 2H, Ar-H), 8.20–8.35 (m, 1H, Ar-H), 12.58 (brs, 1H, NH), 12.94 (brs, 1H, NH); ^13^C-NMR (100 MHz, MeOD) δ 28.74, 29.23, 44.55, 46.94, 50.28, 54.68, 65.24, 100.10, 114.59, 114.97, 121.02, 121.32, 124.21, 128.14, 148.08, 152.32, 153.92, 161.14; MS (ESI): *m*/*z* 509.7 [M + H]^+^; HRMS (ESI): *m*/*z* calcd for C_31_H_36_N_6_O [M + H]^+^ 509.3029; obsd 509.3028.

### 3.3. Antiproliferative Activity

#### 3.3.1. Cell Lines and Cell Culture 

We studied the antiproliferative activity of an initial hit (compound **V)**, and bisbenzimidazole analogs **2a**–**e** in the selected human breast cancer cell lines MDA-MB-231 (ATCC, HTB-26), MDA-MB-468 (ATCC, HTB-132), MCF-7 (ATCC, HTB-22) and the normal breast epithelial cell line, MCF10A, and the ovarian cancer cell lines, A2780, Cis-A2780, and PA-1 (ATCC, CRL 1572). Human breast cancer cell lines and the human ovarian cancer cell line (PA-1) were purchased from ATCC (American Type Culture Collection, Manassas, VA, USA). The cisplatin-sensitive human ovarian carcinoma cell line, A2780, was obtained from Sigma-Aldrich. The cisplatin-resistant human ovarian cell line, Cis-A2780, was produced in our laboratory, as described in the literature [36]. MDA-MB-231, MDA-MB-468, MCF7, and PA-1 were cultured in the MEM medium (Gibco—Life Technologies, Carlsbad, CA, USA). For the the MCF-7 cell line, a final concentration of 0.01 mg/mL human recombinant insulin was added to the media. The normal breast epithelial cell line, MCF-10A, was cultured in complete MEBM (Lonza/Clonetics, Walkersville, MD, USA), as recommended by ATCC. A2780, Cis-A2780 cell lines were cultured in RPMI 1640 medium (Invitrogen, Carlsbad, CA, USA). All media were supplemented with 10% (*v*/*v*) heat-inactivated fetal bovine serum (Biowest LLC, Riverside, MO, USA), 100 U/mL penicillin G and 100 U/mL streptomycin (Sigma Aldrich, St. Louis, MO, USA). Cultures were maintained at 37 °C in a humidified atmosphere containing 5% CO_2_ and were passaged by trypsinization after reaching 80–90% confluence.

#### 3.3.2. Cell Viability Assay

CellTiter 96 AQueous One Solution cell proliferation assay (MTS) [29,30] was used to measure the in vitro cell viability of cells, and the MTS purchased from Promega (Madison, WI, USA). Cells (5 × 10^3^) were seeded into each well of 96-well plates and incubated overnight. Dimethyl Sulfoxide (DMSO) stock solutions of the compounds (**V**, **2a**–**e**) were diluted in corresponding media and exposed to different concentrations for 48 h. All three independent experiments were performed in quadruplicates. Untreated cells were used as a negative control and data were presented as the mean ± standard deviation (S.D.). The absorbances at 492 nm were measured using a Microplate Reader (Synergy HT, Biotek Instruments, Winooski, VT, USA). The compound concentration that inhibited cell growth by 50% of the untreated control (IC_50_) was calculated from the dose response curves constructed by plotting percentages of cell viability versus drug concentrations using nonlinear regression analysis by GraphPad Prism Software 7 (GraphPad Software, San Diego, CA, USA).

#### 3.3.3. Measurement of the Proton (H^+^) Pump Activity

The proton pump activity was assessed by using the AO fluorescence quenching method [35]. Bafilomycin A1 (Baf A1), AO, and nigericin were purchased from Sigma-Aldrich. Hank’s Balanced Salt Solution (HBSS) was purchased from Gibco-Life Technologies (Carlsbad, CA, USA). Human breast cancer MDA-MB-231 (ATCC, HTB-26) cells were washed twice with Hank’s Balanced Salt Solution (HBSS). The cells were re-suspended at a density of 5 × 10^6^ cells per mL in the same buffer and placed on ice. H^+^ uptake was initiated by the addition of MDA-MB-231 cells (10 μL; 0.1 × 10^6^) to pre-warmed HBSS (2.4 mL) containing AO (6 μL; 30 μM) and placed in a cuvette. AO fluorescence was measured at an excitation wavelength of 495 nm and an emission wavelength of 540 nm using the LS-50B Luminescence Spectrometer (Perkin Elmer, Software: FL WinLab, Version 4.00.03). Nigericin (6 μL; 6 μM) was added to collapse the pH gradient and unquench the AO fluorescence (See Supplementary Figure S1: **1a** and **1b**). MDA-MB-231 cells (10 μL; 0.1 × 10^6^) were treated with the standard V-ATPase inhibitor Baf A1 (1 μM), along with compounds **V**, **2d**, and **2e** (10 μM), for 20 min followed by addition of AO (6 μL; 30 μM) for the proton pump activity (See Supplementary Figure S2: **2A**, **2B**, **2C**, and **2D**). A decrease in the fluorescence intensities per min of Baf A1 and the potent compounds (**V**, **2d**, and **2e**) was calculated and is expressed as a percentage of the control (mean ± SD of two independent experiments) (Figure 10). 

## 4. Conclusions

In conclusion, starting from the virtual screening practice using known small molecule V-ATPase inhibitors, we identified a bisbenzimidazole analog (**V**) as an initial hit compound. Synthesis and anticancer screening of a focused set of novel bisbenzimidazole derivatives indicated that they were potent against selected breast and ovarian cancer cell lines in vitro. The selected bisbenzimidazole analogs (**2d** and **2e**) have demonstrated selective potency towards the TNBC cell line, MDA-MB-468. The compound **2e** potently inhibited the proton pump activity, which is largely responsible for the acidification, and it showed selective anticancer activity. To the best of our knowledge, the bisbenzimidazole pharmacophore has been identified as the first V-ATPase inhibitor in its class. Together, our findings demonstrated that the bisbenzimidazole analog (**2e**) could be further developed as an anticancer vacuolar (H^+^)-ATPase inhibitor for the treatment of breast cancer.

## Figures and Tables

**Table 1:** Half maximal inhibitory concentration of novel bisbenzimidazole analogs (**2a**–**e**).

Compd.	IC_50_ ± SD (μM)						
	MDA-MB-468	MDA-MB-231	MCF7	MCF10A	A2780	Cis-A2780	PA-1
**V**	0.72 ± 0.08	1.02 ± 0.08	9.33 ± 0.69	1.14 ± 0.13	3.87 ± 0.09	3.95 ± 0.33	1.70 ± 0.21
**2a**	13.39 ± 0.33	17.56 ± 0.19	49.33 ± 2.76	13.35 ± 0.37	26.49 ± 1.44	25.23 ± 0.32	ND
**2b**	5.83 ± 0.23	4.77 ± 0.43	13.51 ± 2.14	2.95± 0.15	11.80 ± 0.01	12.34 ± 0.02	5.83 ± 0.07
**2c**	1.17 ± 0.36	0.75 ± 0.30	ND	0.81 ± 0.10	12.90 ± 0.42	10.72 ± 0.22	7.78 ± 0.81
**2d**	0.56 ± 0.05	2.44 ± 0.33	22.03 ± 3.9	1.55 ± 0.04	1.97 ± 0.19	3.03 ± 0.18	3.09 ± 0.03
**2e**	0.04 ± 0.02	1.58 ± 0.05	2.02 ± 0.12	1.62 ± 0.14	2.77 ± 0.05	1.34 ± 0.14	2.87 ± 0.15
**Baf A1**	ND	0.026 ± 0.02	ND	0.036 ± 0.04	ND	0.008 ± 0.01	ND

ND: Not Determined.

## Supplementary Materials

The Supplementary Materials are available online.
