# Supplementary material for: Identification of Novel Bisbenzimidazole Derivatives as Anticancer Vacuolar (H+)-ATPase Inhibitors
Source: Molecules. 2017 Sep 16;22(9):1559. doi: 10.3390/molecules22091559 (PMC6151825; doi:10.3390/molecules22091559)
Supplement: Supplementary file 1 [file molecules-22-01559-s001.pdf]

Supporting Information:

Identification of Novel Bisbenzimidazole derivatives as Anticancer Vacuolar (H^+)-ATPase inhibitors†

Renukadevi Patil[1], Arpita Kulshrestha[2], Anjali Tikoo[2], Sara Fleetwood[2], Gajendra Katara[2], Bala Kolli[2], William Seibel[3], Alice Gilman-Sachs[2], Shivaputra A. Patil[1,*] and Kenneth D. Beaman[2,*]

[1] Pharmaceutical Sciences Department, College of Pharmacy, Rosalind Franklin University of Medicine and Science, North Chicago, IL 60064, USA; renukadevi.patil@rosalindfranklin.edu (R.P.); shivaputra.patil@rosalindfranklin.edu (S.A.P.)

[2] Department of Microbiology and Immunology, Chicago Medical School, Rosalind Franklin University of Medicine and Science, North Chicago, IL 60064, USA; arpita.kulshrestha@rosalindfranklin.edu (A.K.); anjali.tikoo@rosalindfranklin.edu (A.T.); sara.fleetwood@rosalindfranklin.edu (S.F.); gajendra.katara@rosalindfranklin.edu (G.K.); bala.kolli@rosalindfranklin.edu (B.K.); alice.gilmansachs@rosalindfranklin.edu (A.G-S.); kenneth.beaman@rosalindfranklin.edu (K.D.B.)

[3] Division of Oncology, Cincinnati Children's Hospital Medical Center, 3333 Burnet Avenue, Cincinnati, Ohio 45229, USA; william.seibel@cchmc.org

* Correspondence: kenneth.beaman@rosalindfranklin.edu ; Tel.: +1-847-578-3449
shivaputra.patil@rosalindfranklin.edu ; Tel.: +1-847-578-8383

1A

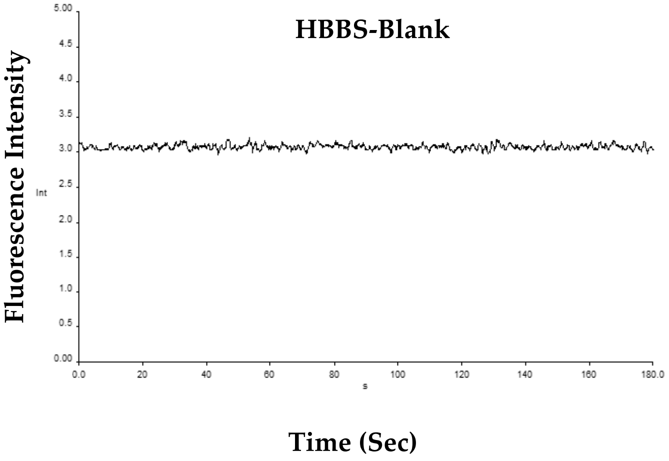

1B

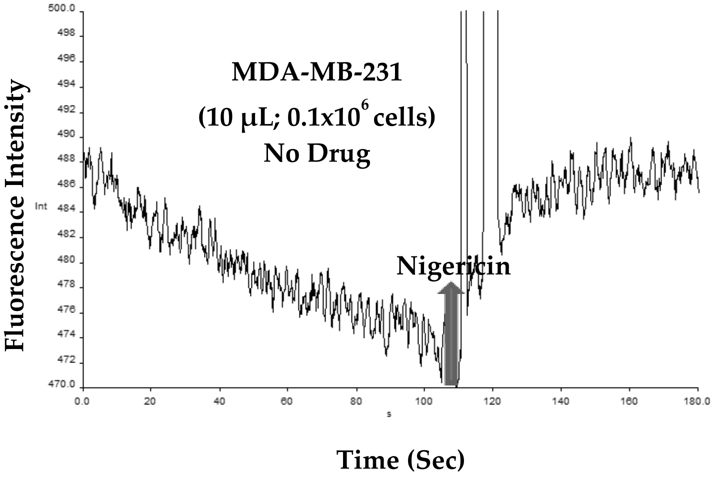

Figure S1. Proton pump activity-Acridine orange (AO) fluorescence quenching method. Fluorescence intensity of Hank's Balanced Salt Solution (HBBS) was measured at an excitation 495 nm and an emission 540 nm (**1A**). Cells (MDA-MB-231) ($0.1x10^6$) were added to HBBS containing AO (30 μM) and AO fluorescence was measured (**1B)**. Upright arrow designates the addition of Nigericin (6 μM) to collapse the proton (H^+) gradient and unquench the AO fluorescence.

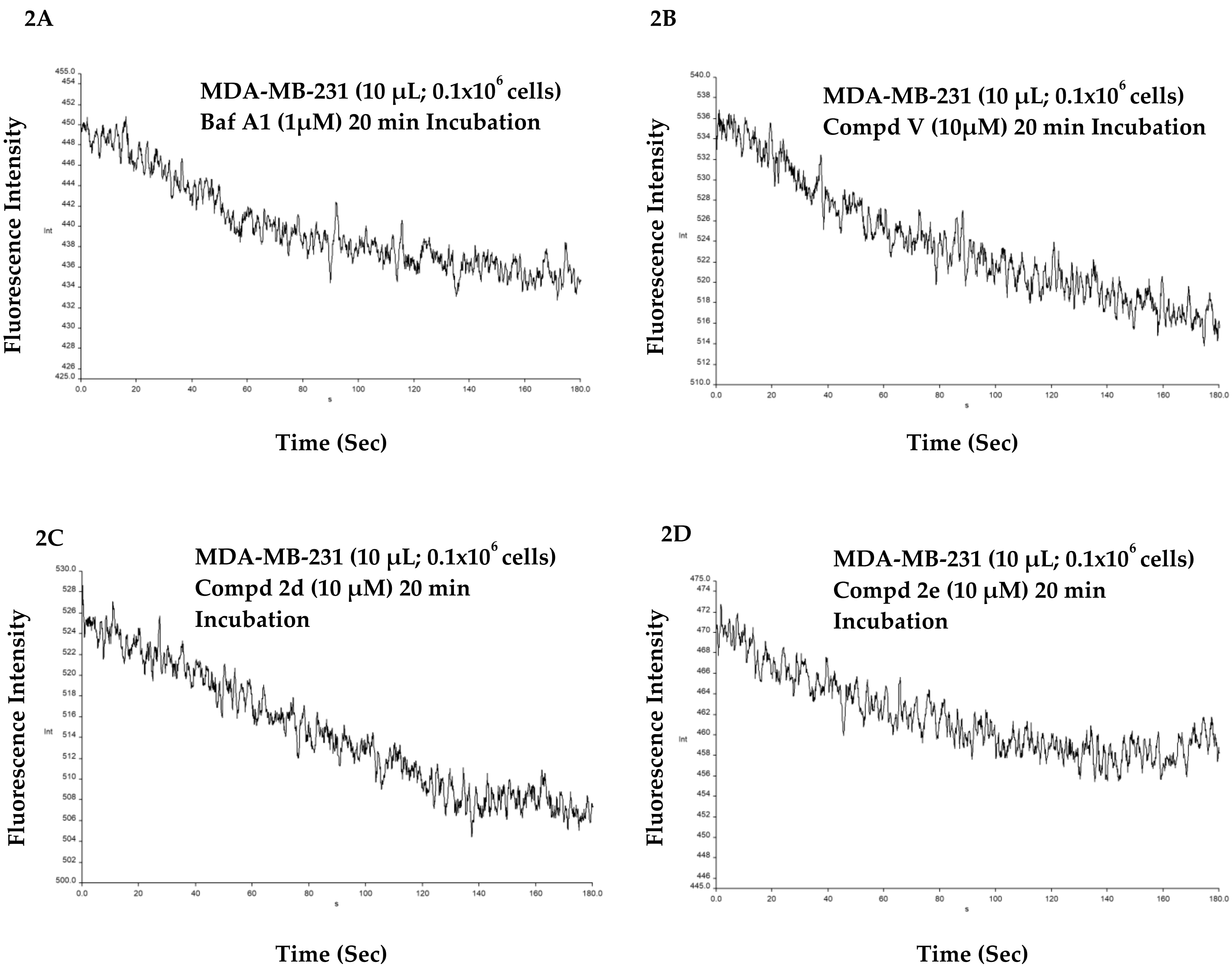

Figure S2. Effect of V-ATPase Inhibitors on Proton pump activity. Cells (MDA-MB-231) (0.1×10^6) were treated with V-ATPase inhibitor Baf A1 (1 µM; Figure **2A**), compounds **V** (10 µM; Figure **2B**), **2d** (10 µM; Figure **2C**), **2e** (10 µM; Figure **2D**) for 20 min followed by addition of AO (30 µM). Fluorescence intensity at an excitation 495 nm and an emission 540 nm was measured.
